# Atrogin-1/MAFbx mRNA expression is regulated by histone deacetylase 1 in rat soleus muscle under hindlimb unloading

**DOI:** 10.1038/s41598-019-46753-0

**Published:** 2019-07-16

**Authors:** Ekaterina P. Mochalova, Svetlana P. Belova, Timur M. Mirzoev, Boris S. Shenkman, Tatiana L. Nemirovskaya

**Affiliations:** Institute of Biomedical Problems, RAS, Khoroshevskoe sh. 76a, 123007 Moscow, Russia

**Keywords:** Physiology, Ubiquitin ligases

## Abstract

It is known that MuRF-1 and atrogin-1/MAFbx mRNA expression is increased in rat soleus muscle under unloading conditions. We aimed to determine the role of histone deacetylase 1 (HDAC1) in the activation of MuRF-1 and MAFbx expression in rat soleus muscle at the early stage of hindlimb suspension (HS). To this end, male Wistar rats (195–215 g) were divided into 3 groups (n = 8/group): control (C), 3-day HS (HS) and 3-day HS + HDAC1 inhibitor CI-994 (1 mg/kg/day) (HS + CI). Protein content and mRNA expression levels of regulatory molecules were determined by Western-blotting and RT-PCR. CI-994 treatment prevented HS-induced increase in HDAC1 nuclear content. As expected, 3-day HS induced a significant upregulation in MAFbx, MuRF-1 and ubiquitin. CI-994 administration resulted in an attenuation of HS-mediated increase in MAFbx and ubiquitin expression levels but did not affect MuRF-1 expression. A decrease in histone acetyltransferase p300 nuclear content in the HS group was prevented by CI-994 administration. There were no significant differences in the content of phosphorylated anabolic signaling molecules between HS group and HS + CI group. Thus, inhibition of HDAC1 prevented a HS-mediated increase in MAFbx and ubiquitin expression, but did not affect MuRF-1 gene expression.

## Introduction

Skeletal muscle is a highly plastic tissue that can adapt its structure and metabolism in response to various conditions. Under unloading conditions skeletal muscles undergo atrophy due to a decrease in protein synthesis and/or an increase in protein breakdown^1–4^. The contribution of ubiquitin-proteasome system (UPS) to protein degradation is especially important, as it accounts for 80–90% of breakdown of all intracellular proteins^5,6^. mRNA expression of the key muscle specific E3 ubiquitin ligases, muscle RING finger-1 (MuRF-1) and muscle atrophy F-box (MAFbx/atrogin-1), serves as a marker for the UPS activity. One of the most investigated mechanism of the regulation of E3 ubiquitin ligases expression is the phosphorylation of transcription factors by protein kinase B (PKB)/Akt^7,8^. Being phosphorylated, transcription factors, such as forkhead box O (FOXO), cannot enter the nucleus and activate the expression of MuRF-1 and MAFbx thereby preventing/attenuating skeletal muscle atrophy. However, in recent studies, we found that phosphorylation of the transcription factor FOXO3 as well as transcription factors of the NF-κB signaling pathway did not always prevent an increase in the expression of E3 ubiquitin ligases in the unloaded skeletal muscle^9–11^. Probably, there are other mechanisms, not related to phosphorylation/dephosphorylation of the transcription factors, which could activate E3 ubiquitin ligases’ genes. To date, it has been proposed that histone deacetylase (HDAC) can interact with various transcription factors, which makes it possible to coordinate and regulate gene expression in skeletal muscles in response to mechanical unloading^12,13^. In addition to gene transcription regulation via histone acetylation/deacetylation, the activity of histone acetyltransferase (HAT) and HDAC can regulate gene expression by changing the acetylation status and function of transcription factors such as FoxO. Currently, there is limited information on specific HDACs that regulate the status of FoxO acetylation in skeletal muscles under normal conditions and those HDAC isoforms which help to reduce acetylation and activation of FoxO during catabolic conditions. In the study by Beharry *et al*. (2014) it was found that HDAC1 can activate FoxO. The authors of that study inhibited the activity of various HDAC isoforms in cell culture and found that HDAC1 isoform is a key regulator of FOXO, which is able to trigger the muscle atrophy program^13^.

We hypothesized that HDAC1 activity is able to control of E3 ubiquitin ligases (MuRF-1 and atrogin-1/MAFbx) mRNA expression in rat soleus muscle at the early stage of unloading. By inhibiting the activity of HDAC1 with CI-994, we aimed to determine whether HDAC1 activity would influence an induction of muscle atrophy program at the early stage of hindlimb unloading. If our hypothesis is correct, inhibition of HDAC1 would lead to downregulation of MuRF-1 and atrogin-1/MAFbx expression and subsequent attenuation of soleus muscle atrophy. As shown earlier, a significant increase in mRNA expression of E3 ubiquitin ligases in soleus muscle is observed at the first day of unloading and reaches the peak expression level by the 3^rd^ day of unloading^14^. This is the reason why, in the present study, a 3-day unloading period has been chosen.

Identification of molecular mechanisms that control the degradation of muscle proteins during mechanical unloading will help to develop a system of pharmacological interventions that could prevent or attenuate skeletal muscle atrophy.

## Materials and Methods

### Ethical approval

All procedures with the animals were approved by the Biomedicine Ethics Committee of the Institute of Biomedical Problems of the Russian Academy of Sciences/Physiology section of the Russian Bioethics Committee (protocol 481, 12 June 2018). All experiments were performed in strict accordance with the guidelines and recommendations in the Guide for the Care and Use of Laboratory Animals of the National Institutes of Health. All efforts were made to minimize the animals’ pain and suffering. Prior to all surgical procedures, the animals were anaesthetized with an intraperitoneal injection of tribromoethanol (240 mg kg−1). The depth of anaesthesia was evaluated by testing the pedal withdrawal reflex (toe and foot pad pinch).

### Animal procedures

All animals were kept at 22 °C; water and food for rodents were available *ad libitum*. Twenty-four 2.5-month-old Wistar male rats were obtained from the certified Nursery for laboratory animals of the Institute of Bioorganic Chemistry of the Russian Academy of Sciences (Pushchino, Moscow region). The rats were randomly assigned to the cage control (C) group (n = 8), hindlimb suspension (HS) group (n = 8), and HS + HDAC1 inhibitor CI-994, Selleckchem, USA, (1 mg/kg daily during 3-day HS i.p. in 2.5% dimethyl sulfoxide (DMSO; Sigma, St Louis, MO, USA) in 0.9% saline, (HS + CI group, n = 8). It was shown earlier that this dose of CI-994 leads to HDAC1 inhibition^15^. Control and HS animals received identical volumes of 2.5% DMSO vehicle. The HS experiment lasted for 3 days. Control animals were housed in groups of three in a temperature- and light-controlled environment (i.e., 12-h light-dark cycle). At the end of the experiment, rats were euthanized with tribromoethanol (10 mg/kg), and soleus muscles were rapidly removed, weighed, and frozen in liquid nitrogen until later analysis. Control animals were processed along with the HS and HS + CI animals.

### Hindlimb suspension protocol

The animals were subjected to unloading conditions using HS^16,17^. A detailed description of the HS protocol can be found in our previous reports^9,10^.

#### Protein extraction and Western blot analysis

A detailed description of protein expression and western-blotting procedures is provided in our previous paper^18^. In brief, samples were loaded and separated on a 10% polyacrylamide gel, followed by transfer to a nitrocellulose membrane (Bio-Rad Laboratories, Hercules, CA, USA), after which membranes were incubated in a blocking buffer (TBS-T: 4% non-fat milk powder; Tris-buffered saline, pH 7.4; and 0.1% Tween 20). The membranes were then incubated with primary and secondary antibodies and washed in TBS-T. The primary antibodies used were HDAC1 (1:500–1000, Cell Signaling, USA, no. 2062), pAkt-473 (1:1000, Cell Signaling, no. 4058), Akt (1:1500, Cell Signaling, no. 9272), p-eEF2 Thr56 (1:1000, Cell Signaling, USA, no. 2331), eEF2 (1:2000, Cell Signaling, USA, no. 2332), calpain-1 (1:1000 Cell Signaling, USA, no. 2526); p-FoxO3 Ser253 (1:1000, Santa Cruz, USA, sc101683), FoxO3 (1:1000, Thermo Fisher Scientific, USA, # PA5-20973), acetyl-Histone H3 (1:500, «Merk», USA, no. 06-599), HAT P300 (1:2000, «Abcam», USA, ab231010), lamin B1 (1:500, Abcam, ab16048) and glyceraldehyde-3-phosphate dehydrogenase (GAPDH, 1:10000; Applied Biological Materials Inc., Richmond, British Columbia, Canada, no. G041). Secondary antibodies (1:4000) to rabbit or mouse immunoglobulins were from Santa Cruz Biotechnology, USA. Protein bands were detected and quantified using Immun-Star HRP Chemiluminescent kit (Bio-Rad Laboratories, USA) and C-DiGit Blot Scanner (LI-COR Biotechnology, Lincoln, NE, USA).

### RNA analysis

RT-PCR analysis was performed as reported previously^10^. Briefly, total RNA was extracted from 10 mg frozen soleus muscle using the RNeasy Micro Kit (Qiagen, Hilden, Germany). RNA concentration was determined at 260 nm. For each target mRNA, 1 µl cDNA was amplified in a 25 µl SYBR Green PCR reaction containing 1x Quantitect SYBR Green Master Mix (Syntol) and 10 µM of each primer: 5′-CTACGATGTTGCAGCCAAGA-3′ and 5′-GGCAGTCGAG AAGTCCAGTC-3′ for MAFbx; 5′-GCCAATTTGGTGCTTTTTGT-3′ and 5′-AAATTCAGT CCTCTCCCCGT-3′ for MuRF-1; 5′-CACCAAGAAGGTCAAACAGGA-3′ and 5′-GCAAGA ACTTTATTCAAAGTGCAA-3′ for ubiquitin, 5′-ACGGCAAGTTCAACGG CACAGTCAA-3′ and 5′-GCTTTCCAGAGGGGCCATCCACA-3′ for GAPDH; 5′-TCATGAAGTGTGACGTT GACATCC-3′ and 5′-GTAAAACGCAGCTCAGTAACAGTC-3′ for β-actin. Samples to be compared were run under similar conditions (template amounts, duration of PCR cycles). The amplification was monitored in a real time using the iQ5 multicolor real-time PCR detection system (Bio-Rad Laboratories, USA). β-Actin and GAPDH were used as the housekeeping genes.

### Statistical analysis

All PCR data are expressed as median and interquartile range (0.25–0.75). Statistical analysis was performed using the REST 2009 v.2.0.12 (Qiagen, Germany) and Origin Pro v.8.0 (OriginLab Corp., Northampton, MA, USA) programs. All Western blot data are expressed as means ± SE. significant differences between groups were statistically analyzed using 2-way ANOVA followed by Tukey’s test. When normality testing failed, data were analyzed by nonparametric methods (Kruskal-Wallis ANOVA followed by Dunnett’s test). Differences with values of P < 0.05 were considered to be statistically significant.

## Results

**Figure 1:**
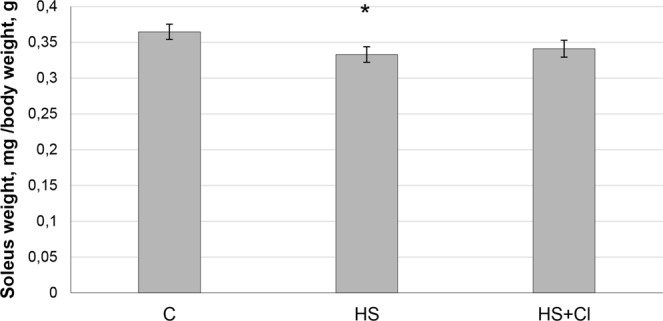
Soleus weight-to-body weight ratio. С – control, HS – 3-day hindlimb suspension + placebo, HS + CI – 3-day hindlimb suspension + CI-994 (HDAC1 inhibitor). All values are means ± SE, n = 8/group. ^*^significant difference from control (p < 0.05).

**Figure 2:**
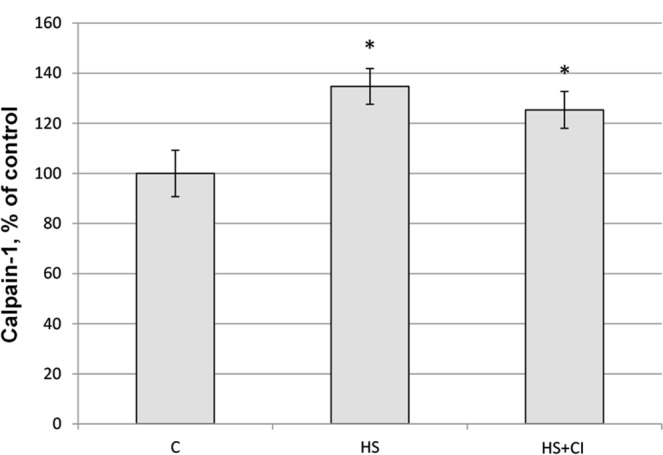
The content of calpain-1 in rat soleus muscle. С – control, HS – 3-day hindlimb suspension + placebo, HS + CI – 3-day hindlimb suspension + CI-994 (HDAC1 inhibitor). All values are means ± SE, n = 8/group. ^*^significant difference from control (p < 0.05).

**Figure 3:**
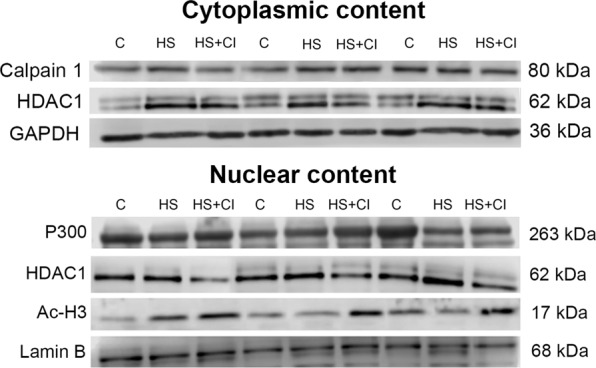
Representative western blots for Calpain-1, HDAC1, P300, Ac-H3, GAPDH and Lamin B in cytoplasmic and nuclear fractions.

The weight of experimental rats was 195–215 g at the end of the experiment and did not significantly differ between the groups. We found a significant 10% decrease (p < 0.05) in soleus weight-to-body weight ratio in the HS group compared to the C group (Fig. 1). This parameter in the HS + CI group did not significantly differ from the C group. Along with the difference in soleus weight-to-body weight ratio we found a significant difference in the mRNA expression of ubiquitin between the HS and HS + CI groups (Table 1). It is interesting to note that in the unloading group with HDAC1 inhibition (HS + CI) ubiquitin mRNA expression did not differ from the control level, but was significantly lower than that in the HS group. Despite the differences in ubiquitin expression, calpain-1 content in both unloaded groups was increased (p < 0.05) relative to the control group (Figs 2 and 3).

**Table 1:** Relative mRNA expression levels of MuRF-1, Atrogin-1/MAFbx and ubiquitin (Ub) in rat soleus muscle.

	С	HS	HS + CI
MuRF-1	1.00 (0.90–1.02)	1.67 (1.64–1.79)*	1.57 (1.53–1.72)*
MAFbx	1.00 (0.96–1.12)	2.12 (1.83–2.81)*	1.67 (1.60–1.83)*#
Ub	1.00 (0.92–2.15)	4.21 (3.86–4.63)*	2.31 (1.69–3.43)#

С – control, HS – 3-day hindlimb suspension + placebo, HS + CI – 3-day hindlimb suspension + CI-994 (HDAC1 inhibitor). The data are expressed as median and interquartile range (0.25–0.75), n = 8/group. *significant difference from control (p < 0.05), ^#^significant difference from the HS group (p < 0.05).

**Figure 4:**
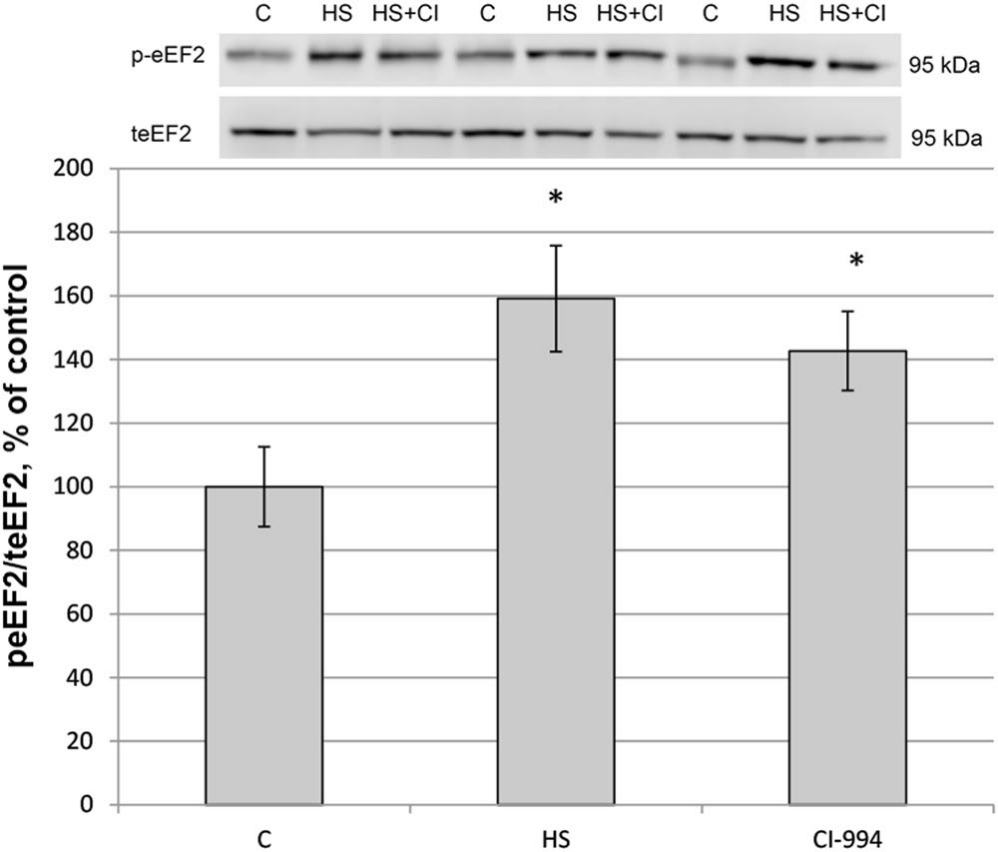
The level of eEF2 phosphorylation in rat soleus muscle. С – control, HS – 3-day hindlimb suspension + placebo, HS + CI – 3-day hindlimb suspension + CI-994 (HDAC1 inhibitor). All values are means ± SE, n = 8/group. *significant difference from control (p < 0.05).

A significant increase in phosphorylated elongation factor 2 (eEF2)/total eEF2 ratio was observed in the HS and HS + CI groups as compared with the C group (Fig. 4).

The expression levels of content of E3 ubiquitin ligases MuRF1 and atrogin-1/MAFbx were significantly increased in both unloaded groups (HS and HS + CI) relative to the C group (Table 1). At the same time, in the HS + CI group the level of Atrogin - 1/MAFbx mRNA expression was significantly lower than in the HS group (Table 1), whereas MuRF1 mRNA expression levels did not show any differences between the two unloaded groups (Table 1).

**Figure 5:**
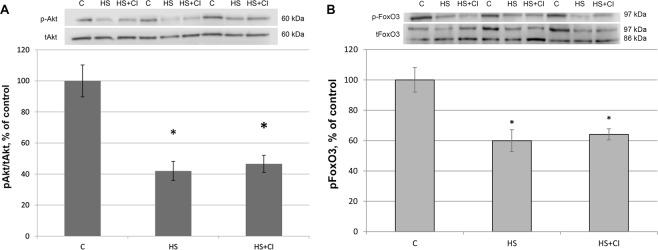
The content of phosphorylated Akt (S473) (A) and FoxO3 (S253) (B) in rat soleus muscle. С –control, HS – 3-day hindlimb suspension + placebo, HS + CI – 3-day hindlimb suspension + CI-994 (HDAC1 inhibitor). All values are means ± SE, n = 8/group. *significant difference from control (p < 0.05).

The level of FOXO3 phosphorylation was equally reduced in both unloaded groups relative to the C group (Fig. 5B). The same pattern of phosphorylation was observed for Akt, which is known to phosphorylate FOXO3 (Fig. 5A).

**Figure 6:**
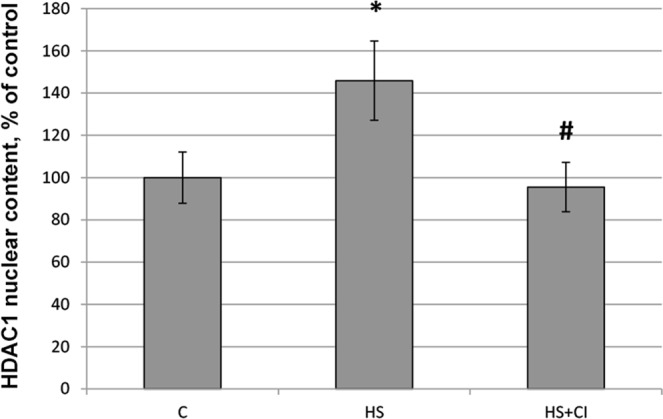
Nuclear content of HDAC1 in rat soleus muscle. С – control, HS – 3-day hindlimb suspension + placebo, HS + CI – 3-day hindlimb suspension + CI-994 (HDAC1 inhibitor). All values are means ± SE, n = 8/group. ^*^significant difference from control (p < 0.05), ^#^significant difference from the HS group (p < 0.05).

**Figure 7:**
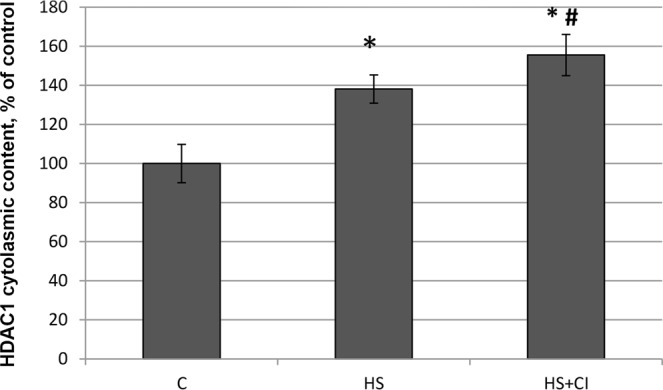
Cytoplasmic content of  HDAC1 in rat soleus muscle. С – control, HS – 3-day hindlimb suspension + placebo, HS + CI – 3-day hindlimb suspension + CI-994 (HDAC1 inhibitor). All values are means ± SE, n = 8/group. ^*^significant difference from control (p < 0.05), ^#^significant difference from the HS group (p < 0.05).

We found that the content of HDAC1 in the nuclear fraction was significantly increased (p < 0.05) only in the HS group but not in the group with HDAC1 inhibitor administration (HS + CI) (Figs 3 and 6). At the same time, in the cytoplasmic fraction the content of HDAC1 was increased (p < 0.05) in both unloaded groups (HS and HS + CI) in comparison with the C group (Figs 3 and 7).

**Figure 8:**
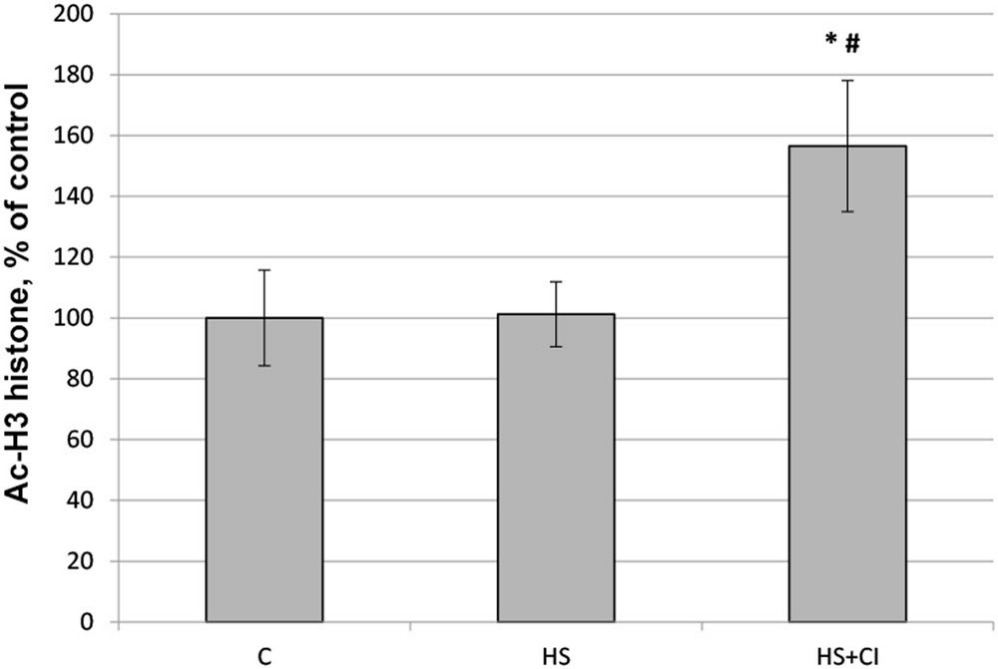
Nuclear content of Ac-H3 in rat soleus muscle. С – control, HS – 3-day hindlimb suspension + placebo, HS + CI – 3-day hindlimb suspension + CI-994 (HDAC1 inhibitor). All values are means ± SE, n = 8/group. *significant difference from control (p < 0.05), ^#^significant difference from the HS group (p < 0.05).

**Figure 9:**
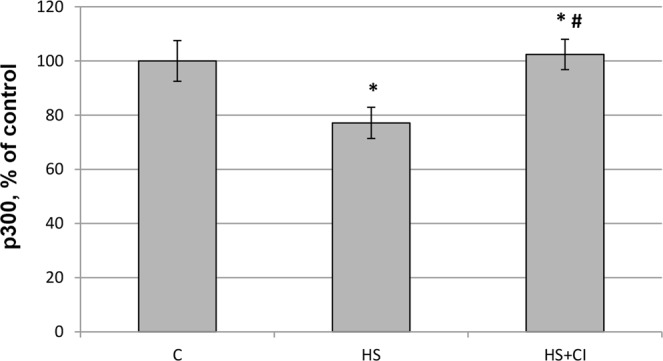
Nuclear content of histone acetyltransferase p300 in rat soleus muscle. С – control, HS – 3-day hindlimb suspension + placebo, HS + CI – 3-day hindlimb suspension + CI-994 (HDAC1 inhibitor). All values are means ± SE, n = 8/group. *significant difference from control (p < 0.05), ^#^significant difference from the HS group (p < 0.05).

The content of histone H3 acetylated on an N-terminal tail (Ac-H3) in the nucleus (detected with anti-acetyl-Histone H3 antibody) was sharply increased in the HS + CI group relative to both C and HS groups (Figs 3 and 8). At the same time, the nuclear content of histone acetyltransferase p300 was reduced only in the HS group vs. the C group (Figs 3 and 9). Administration of HDAC1 inhibitor during 3-day HS prevented the decrease in p300 nuclear content in rat soleus muscle (Figs 3 and 9).

## Discussion

Previous report suggests that a decrease in skeletal muscle mass can occur during the early stages (2–4 days) of unloading^19^. The effect of HDAC1 inhibition on the soleus weight-to-body weight ratio that was observed in the present study occurred at the early stage of HS (3 days).

In skeletal muscle, the UPS is responsible for the breakdown of sarcomeric proteins with changes in motor activity^20^. Increased ubiquitination contributes to the development of muscle atrophy. Judging by the ubiquitin expression (Table 1), it appears that in the HS group this process is much faster than in the other two groups, however CI-994 administration slowed it down. Calpains can serve as a starting point, triggering further ubiquitination of proteins during mechanical unloading of skeletal muscles^11^. At the same time, calpain and UPS systems do not always work in concert during muscle atrophy^21^. We found that calpain expression in the HS and HS + CI groups was significantly higher than that in the C group (Figs 2 and 3), therefore it can be assumed that the inhibition of HDAC1 does not affect the calpain-1 content during HS, and is unlikely to be related to the differences in the rate of atrophy between the HS and HS + CI groups.

It is known that phosphorylation of eEF2 by elongation factor 2 kinase (eEF2K) kinase down-regulates translation elongation^22^. Since the level of eEF2 phosphorylation was significantly increased in both unloaded groups vs. the C group (Fig. 4), it can be assumed that HDAC1 is not involved in the regulation of this process.

A reduction in skeletal muscle mass is significantly associated with increased expression of E3 ubiquitin ligases^23^. The contribution of the UPS to protein degradation is particularly significant, because it plays a central role in the selective degradation of intracellular proteins^5^. In both unloaded groups the expression of E3 ligases atrogin-1/MAFbx and MuRF1 was increased (Table 1). These muscle-specific E3-ligases are rapidly activated during mechanical unloading^4,24^. In the HS + CI group the increase in Atrogin-1/MAFbx mRNA expression was significantly lower than that in the HS group (Table 1) whereas the MuRF-1 expression in the HS + CI group was the same as in the HS group. It is believed that these two E3 ligases play a different role in the atrophic process. MuRF-1 is involved in the breakdown of myofibrillar proteins^25^, while Atrogin-1/MAFbx is able to control both the processes associated with the breakdown of cytoskeletal proteins as well as processes associated with protein synthesis^26^. Our findings suggest that HDAC1 is involved in the regulation of Atrogin-1/MAFbx mRNA expression. It is known that the expression of atrogin-1/MAFbx and MuRF-1 during muscle unloading is controlled by the transcription factor FOXO1/3^27^. The level of FOXO phosphorylation/dephosphorylation is frequently investigated since FOXO1/3 can be involved in the regulation of E3 ligases; however, the level of FOXO3 phosphorylation in our study was equally reduced in both unloaded groups (Fig. 5B). In turn, FOXO3 is phosphorylated and regulated by Akt, and Akt phosphorylation level was reduced in the HS and HS + CI groups (Fig. 5A). It can be assumed that in skeletal muscle there is another mechanism that regulates the expression of atrogin-1/MAFbx independent of phosphorylation/dephosphorylation of FOXO3a. We found that HDAC1 nuclear content was increased only in the HS group (p < 0.05), but not in the group with HDAC1 inhibitor (HS + CI) (Figs 3 and 6). It appears that CI-994 treatment prevented HDAC1 traffic to the nucleus, resulting in a slight accumulation of HDAC1 in the cytoplasm (Figs 3 and 7). Earlier, Moresi *et al*. (2010) showed that accumulation of HDAC5 in the nucleus can lead to an increase in the expression of E3 ubiquitin ligases^12^. It is known that nuclear-cytoplasmic traffic of HDAC1 can control the activity of promoters of genes encoding E3 ubiquitin ligases^13,28^. It has been also shown that, under basal conditions, the activity of FoxO can be repressed by reversible acetylation of lysine. This is due to the deacetylase activity of HDAC1, as deacetylation of FOXO3a^29^ is required to activate the expression of MuRF-1 and atrogin-1/MAFbx. HDACs are able to interact with various transcription factors, which make it possible to coordinate and regulate gene expression in skeletal muscles in response to mechanical unloading. In addition to the gene transcription regulation via acetylation of histones, HAT and HDAC catalytic activity can be involved in the regulation of gene expression by changing the acetylation status of various transcription factors including FoxO. Currently, there is a lack of data on specific HDACs that could be involved in the regulation of FoxO acetylation in skeletal muscles under both normal and unloading conditions. Beharry *et al*. (2014) found that HDAC1 is able to activate FoxO^13^. The authors inhibited the work of various histone deacetylases in cell culture and found that HDAC1 is a key regulator of FOXO, which can trigger the muscle atrophy program. The aim of our study was to check whether HDAC1 can control the expression of E3 ligases (MuRF-1 and atrogin-1/MAFbx) in rat soleus muscle under hindlimb unloading. Using HDAC1 inhibitor CI-994, we have determined the relationship between HDAC1 deacetylase activity and the induction of the muscle atrophy program. Since a significant expression of E3 ligases is observed at the early stage of skeletal muscle unloading^14^, we conducted a study with a 3-day HS. Unfortunately, specific antibodies to the site of FOXO3a acetylation are not available. In the present study we determined the content of Ac-H3 and revealed a sharp increase in its content in the group with inhibition of HDAC1 during HS (HS + CI group) (Figs 3 and 8). Bertaggia *et al*. (2012) have shown that FOXO3a interacts with and is acetylated by HAT^29^. A decrease in HAT activity in skeletal muscle induces FoxO transcriptional activity, while an increase in HAT activity prevents nuclear localization of FoxO, thereby decreasing its transcriptional activity towards target genes^13^. We found a decrease in p300 nuclear content in the HS group, but not in the HS + CI group as compared with the control values (Figs 3 and 9). The p300 binding protein appears to interact with numerous transcription factors. In the paper by Senf *et al*. (2011) it was shown that a decrease in p300 activity leads to an increase in the expression of MAFbx/atrogin-1 under conditions of denervation-induced atrophy^30^. These authors indicate that p300 is able to reduce the transcriptional activity of FOXO3 by preventing its nuclear localization through acetylation, and thereby influence the expression of MAFbx/atrogin-1. It should be noted that a decrease in p300 in our experiment was observed only in the HS group (without HDAC inhibitor), in which Atrogin-1 expression was the highest. It can be assumed that, in our case, a decrease in the Atrogin-1 mRNA expression could occur due to a decrease in the transcriptional activity of FOXO3a because of an increase in its acetylation. In skeletal muscle, HDACs are implicated in the control of gene expression via the regulation of histone acetylation, resulting in chromatin modifications and subsequent activation or repression of transcription^31^. Probably, HDAC1 inhibition could lead to reduced FOXO3a transcriptional activity resulting in the increase in MAFbx/atrogin-1 mRNA expression.

## Conclusion

We have shown for the first time that (i) HDAC1 controls the expression of E3 ligase Atrogin-1/MAFbx in rat soleus muscle under mechanical unloading and (ii) inhibition of HDAC1 can attenuate skeletal muscle atrophy. It is not excluded that the control of Atrogin-1 mRNA expression (along with phosphorylation/dephosphorylation of FOXO3a) is carried out by its acetylation/deacetylation upon 3-day hindlimb unloading.
